# Msl2 Is a Novel Component of the Vertebrate DNA Damage Response

**DOI:** 10.1371/journal.pone.0068549

**Published:** 2013-07-09

**Authors:** Zheng Lai, Simona Moravcová, Yvan Canitrot, Lukasz P. Andrzejewski, Dervla M. Walshe, Stephen Rea

**Affiliations:** 1 Centre for Chromosome Biology, School of Natural Sciences, National University of Ireland, Galway, University Road, Galway, Ireland; 2 Université de Toulouse, UPS, CNRS, Toulouse, France; University of Massachusetts Medical School, United States of America

## Abstract

hMSL2 (male-specific lethal 2, human) is a RING finger protein with ubiquitin ligase activity. Although it has been shown to target histone H2B at lysine 34 and p53 at lysine 351, suggesting roles in transcription regulation and apoptosis, its function in these and other processes remains poorly defined. To further characterize this protein, we have disrupted the *Msl2* gene in chicken DT40 cells. *Msl2^−/−^* cells are viable, with minor growth defects. Biochemical analysis of the chromatin in these cells revealed aberrations in the levels of several histone modifications involved in DNA damage response pathways. DNA repair assays show that both *Msl2^−/−^* chicken cells and hMSL2-depleted human cells have defects in non-homologous end joining (NHEJ) repair. DNA damage assays also demonstrate that both Msl2 and hMSL2 proteins are modified and stabilized shortly after induction of DNA damage. Moreover, hMSL2 mediates modification, presumably ubiquitylation, of a key DNA repair mediator 53BP1 at lysine 1690. Similarly, hMSL1 and hMOF (males absent on the first) are modified in the presence of hMSL2 shortly after DNA damage. These data identify a novel role for Msl2/hMSL2 in the cellular response to DNA damage. The kinetics of its stabilization suggests a function early in the NHEJ repair pathway. Moreover, Msl2 plays a role in maintaining normal histone modification profiles, which may also contribute to the DNA damage response.

## Introduction

DNA double strand breaks (DSBs) are a particularly dangerous form of damage, as their inaccurate repair or lack of repair can result in mutations or chromosomal translocations leading to cancer. DSBs can be repaired by either of two processes: non-homologous end joining (NHEJ) or homologous recombination (HR) [1], [2]. HR repair occurs in S- and G2-phases of the cell cycle, when it can use the undamaged nearby homologous sister chromatid’s DNA as a template to faithfully repair the break. NHEJ occurs throughout the cell cycle, is faster than HR, and results in ligation of the two broken DNA fragments [1], [2]. Both pathways comprise a series of stages that involve a large and growing number of proteins; sensors first detect that there is a double-stranded break in the DNA. Next, mediators and transducers get recruited to damaged chromatin, where they accumulate. The signal is amplified and passed on to effector proteins. These effectors enable cell cycle arrest and the repair of the broken DNA [2], [3], [4].

The choice of which pathway a cell takes to repair a DSB is dependent on the stage of the cell cycle and the complexity of the damage, and is crucial to the damaged cell. Perturbation in the balance between HR and NHEJ can result in disease, but can also be exploited in the treatment of cancer [1]. One of the proteins regulating this choice is 53BP1 (p53 binding protein 1). It can inhibit DNA resection, and thus HR repair, promoting the NHEJ pathway [5], [6], [7]. Following DNA damage it gets recruited to and accumulates at chromatin surrounding the damage site through interaction with methylated histone residues (H3K79me2, H4K20me2) via its tandem tudor domains [8], [9], [10], [11], and through interaction with the damage mediator protein MDC1 via a central core region. Once 53BP1 accumulates it is involved in recruitment of other DDR proteins, facilitating accessibility to the chromatin [12], or otherwise promoting repair [6], [7]. However, it is still unclear how the enzymes mediating these 53BP1-recruiting modifications are themselves regulated in response to DNA damage.

MSL2 (male-specific lethal 2) was originally identified in the fruitfly, *Drosophila melanogaster*, in genetic screens for mutants causing male-specific lethality. Such genes were implicated in dosage compensation, a process that ensures equal amounts of X-chromosomal gene expression between males and females with unequal numbers of this sex chromosome (reviewed in [13], [14]). MSL2 was subsequently shown to be a pivotal participant in this process. Its expression is achieved only in male flies and is required for the formation of the MSL complex (also known as the dosage compensation complex) [15], and its initial recruitment to the male X chromosome [16], whence it mediates the 2-fold increase in transcription of X-linked genes [13], [14]. It was recently shown that MSL2 can ubiquitylate MSL1, as well as MSL3 and MOF [17], [18]. This ubiquitylation can target these proteins for proteasome-mediated degradation to control MSL complex stoichiometry, but is also proposed to regulate their recruitment to specific chromatin domains [18].

Human orthologues of these MSL proteins exist and they are found in an evolutionary conserved human MSL complex, also known as the hMOF (Males absent on the first, human) complex [19], [20], [21]. Direct studies on hMSL2 are few; one study found that when overexpressed, it is able to mono-ubiquitylate p53 at lysine 351. This targets p53 for export to the cytoplasm where it induces mitochondrial-dependent apoptosis [22], [23]. Mutation of this residue has been reported in a cisplatin-resistant ovarian carcinoma cell line [23]. A second study has recently shown that hMSL2 in tandem with hMSL1 is able to ubiquitylate histone H2B on lysine 34, and that this H2BK34ub directly regulates methylation of H3K4 and H3K79 by trans-tail crosstalk to promote transcription [24].

Other components of the human MSL/MOF complex include hMOF, hMSL1, hMSL3, and NUP153 (Nucleoprotein 153) [19], [20], [21]. hMOF is involved in transcriptional regulation [19], [25], is required for embryogenesis [26], is downregulated in several cancers [27], and importantly here, is known to participate in the DNA damage response (DDR) [28], [29], [30], [31]. hMOF is responsible for the acetylation of histone H4 at lysine 16 (H4K16ac) [21], [26], [28], [30]. In the DDR, this modification is required for the recruitment of MDC1 (Mediator of DNA damage checkpoint 1) [28], [29]. hMOF has also been proposed to regulate ATM (Ataxia telangiectasia mutated) function following DNA damage [31]. Moreover, it can acetylate p53 in response to high levels of damage promoting p53-dependent transcription of pro-apoptotic genes [32]. Interestingly, hMSL1 is known to influence hMOF’s H4K16 acetylation activity [21], [33], and it has been shown to co-immunoprecipitate with the DNA repair mediator, 53BP1 [33].

The aim of this study was to determine the cellular function(s) of MSL2 in higher eukaryotes, and to investigate whether this function involves MSL2’s interaction with other MSL proteins.

Knock-out of chicken *Msl2* in DT40 cells has revealed an important role of Msl2 in the DNA damage response. We found that Msl2 is required for normal levels of several histone modifications involved in the DDR, including those that recruit 53BP1. Msl2 is also required for full NHEJ efficiency, as is the human orthologue hMSL2. Both human and chicken proteins are rapidly stabilized in response to DNA damage, and hMSL2 mediates the possible ubiquitylation of 53BP1, hMSL1 and hMOF. These data define Msl2/hMSL2 as a novel player in the NHEJ pathway, acting early in the DDR, and upstream of the modifications and proteins that recruit 53BP1.

## Results

### Msl2 Knockouts are viable with Minor Growth Defects

**Figure 1:**
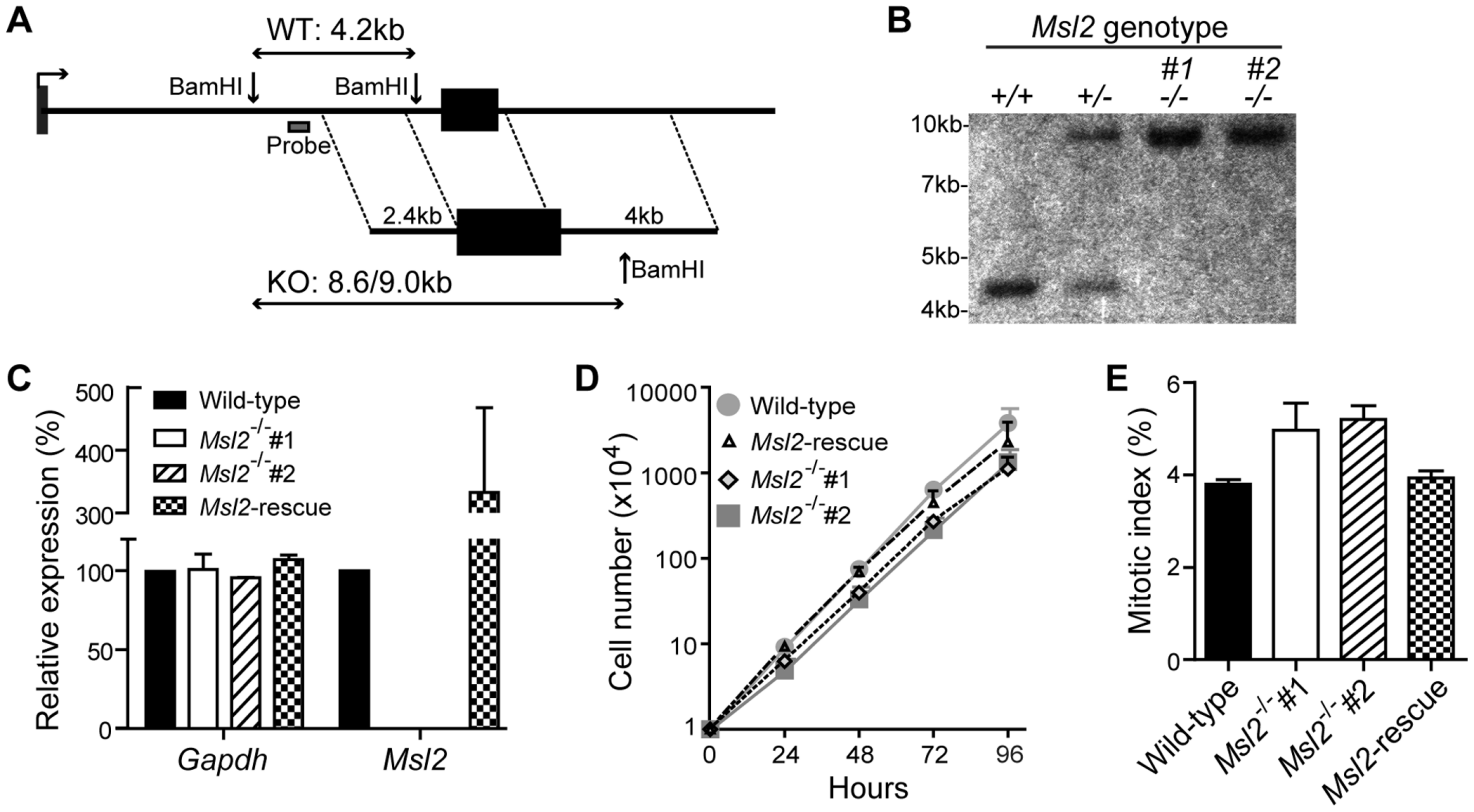
*Msl2* knockout cells are viable with a minor growth defect. (**A**) Schematic depicting *Msl2* locus and targeting strategy: A targeting cassette with 2.4 and 4 Kb homology arms targets the second of *Msl2’s* two exons (black boxes). Successful targeting (KO) results in an 8.6/9 Kb BamH1 digestion fragment depending on puromycin (Pur)/Blasticidin (Bla) resistance of cassette. (**B**) Southern blot confirmation of targeting. (**C**) Q-PCR derived expression levels of *Gapdh* and *Msl2* mRNA in wild-type, *Msl2^−/−^*, and *Msl2*-rescue cell lines. Expression is normalized to *ß-actin* and compared to wild-type levels. Error bars represent standard deviation (n = 3). (**D**) Proliferation analysis of wild-type, *Msl2^−/−^*, and *Msl2*-rescue cell lines. Error bars represent standard deviation (n = 4). (**E**) Mitotic index as determined by percentage of H3S10ph positive cells according to flow cytometry analysis. Error bars represent standard deviation (n = 3).

To determine the function of Msl2 in vertebrates we targeted the chicken gene, *Msl2* for disruption in DT40 cells. Using available database information we found only one *Msl2* gene in chicken. Located on chromosome 9, this 4.3 Kb gene comprises 2 exons (Figure 1A), as in humans. The encoded 579 amino acid protein is highly conserved between chicken and human, with a sequence identity of 83% (Supplementary Figure 1). PCR with primers designed using the chicken database, confirmed expression of *Msl2* mRNA in DT40 cells (data not shown). To disrupt Msl2 function, we used genomic PCR to generate targeting constructs that would delete the larger second exon encoding 92% of the protein (Figure 1A).

Successful targeting of both alleles was confirmed by Southern blotting analysis (Figure 1B) and loss of expression of *Msl2* was confirmed by quantitative real time PCR (Q-PCR)(Figure 1C). We used two independent clones; *Msl2* ^−/−^ #1 and *Msl2* ^−/−^ #2 in the following analysis. To ensure that any phenotypes observed in the knock-out cell lines are due to loss of Msl2, we created a cell line expressing HA-2xFlag-tagged Msl2 (HA2F-Msl2) in the *Msl2* ^−/−^ #2 background (Msl2-rescue). According to the amount of *Msl2* mRNA produced, the rescue cells express approximately 3.3 times the level of Msl2 as wild-type cells (Figure 1C).

Our first observation was that the *Msl2* ^−/−^ cells were viable, with morphology similar to wild-type (not shown), demonstrating that *Msl2* is not an essential gene in DT40 cells. We then examined the proliferative ability of *Msl2* ^−/−^ cells. We found that cells lacking Msl2 proliferate more slowly than wild-type cells (Figure 1D). Wild-type cells proliferated with a doubling time of 8.05 hours whereas *Msl2* ^−/−^ #1 and #2 took 9.18 and 9.62 hours respectively, a delay of approximately 20%. The rescue cells displayed a recovery of this delay, having a doubling time of 7.95 hours. We observed an increase in the mitotic index in the *Msl2* ^−/−^ cells (5%) compared to wild-type (4%), and this was reduced to wild-type levels in the Msl2-rescue line (Figure 1E). This increase may partially explain the defect in proliferation.

### Histone Modifications are Perturbed in Msl2 Knockout Cells

The exact cause of this growth defect and delay is unclear. As mentioned above, human MSL2 is a component of the hMSL complex with hMOF [20], [21], and depletion of hMOF causes a number of phenotypes including a G2/M arrest reminiscent of the delay we observed, as well as DNA repair defects [21], [28], [30]. The Becker lab has recently shown that *Drosophila* MSL2 ubiquitylates MOF, and other MSL proteins, controlling the stoiciometry of the complex [18]. We therefore wanted to determine whether chicken Mof was affected by loss of Msl2, and whether these knockout cells had similar defects.

**Figure 2:**
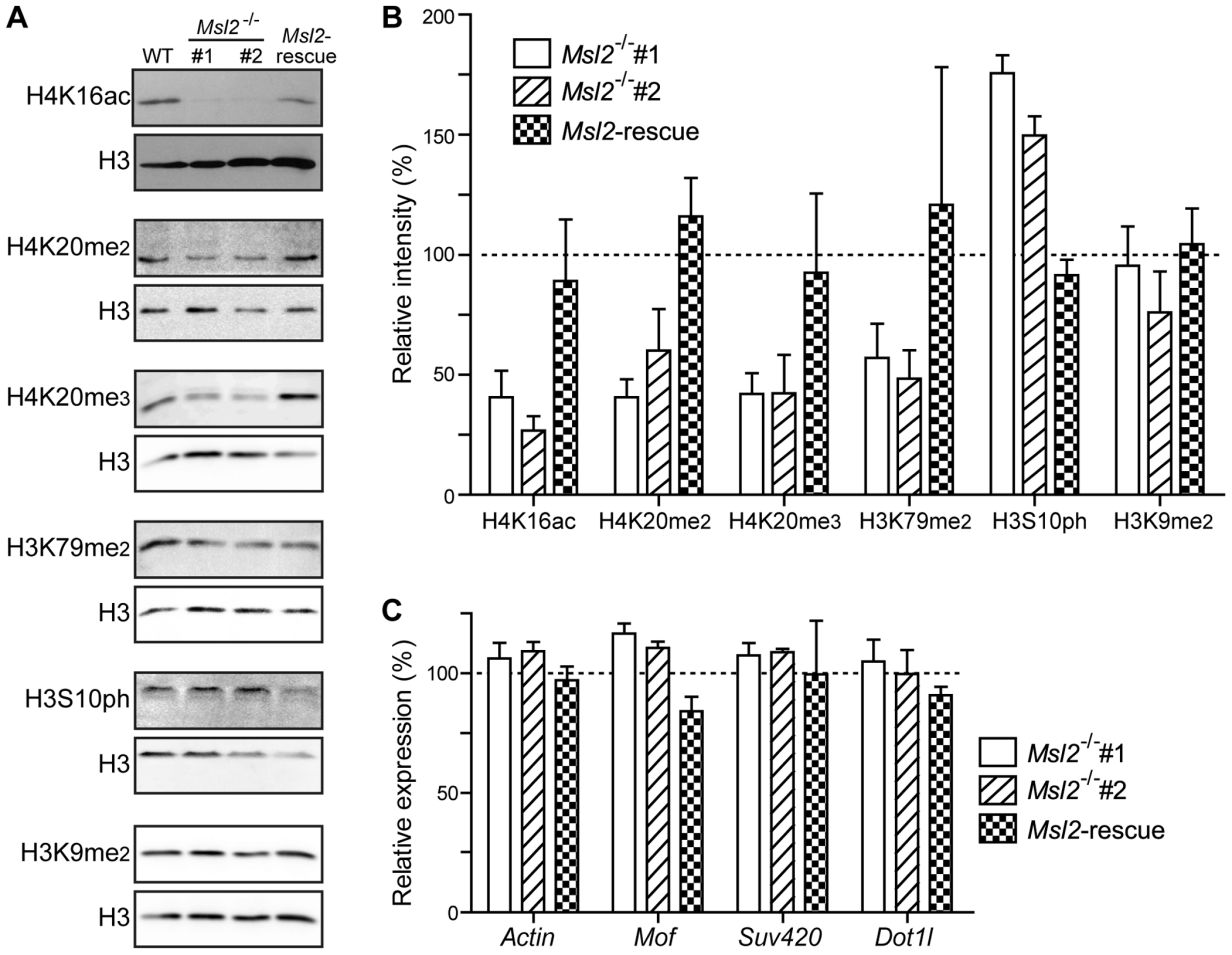
Histone modifications are perturbed by loss of Msl2. (**A**) Representative immunoblot analysis of nuclear extracts prepared from wild-type, *Msl2^−/−^*, and *Msl2*-rescue cell lines. Panels were probed with the antibodies indicated. (**B**) Quantification of (A). Mean expression levels of the various modifications in the cell lines were quantified and expressed relative to those in wild-type cells after normalization to H3 levels. Error bars represent standard deviation (n ≥3). (**C**) Q-PCR showing expression levels of the genes indicated. Expression levels in the cell lines are expressed relative to those in wild-type cells, following normalization to *Gapdh*.

In the absence of a functional antibody to chicken Mof protein, we looked at the acetylation levels of one of its substrates; lysine 16 on histone H4 (H4K16ac) for which it is responsible [21], [30], [34]. We prepared nuclear extracts and quantified the level of H4K16ac in wild-type or *Msl2* knockout cells by immuno-blotting analysis. We found that the level of this modification in *Msl2* ^−/−^ cells was reduced to ∼40% that in wild-type (Figure 2A, B). This decrease in H4K16 acetylation returned to normal levels in the Msl2-rescue cell line. This suggests that Msl2 regulates the activity of the Mof enzyme, and/or the stability of the complex.

In addition we looked at several other histone modifications; di- and tri-methylation of lysine 20 on histone H4 (H4K20me2/3), di-methylation of lysine 79 on histone H3 (H3K79me2), phosphorylation of serine 10 on histone H3 (H3S10ph), and di-methylation of lysine 9 on histone H3 (H3K9me2). Interestingly, we found that the levels of H4K20me2, H4K20me3 and H3K79me2 were reduced to 50% or less than that in wild-type cells. In contrast, H3S10ph levels increased significantly (Figure 2A, B), in keeping with the mitotic index data (Figure 1E). H3K9me2 levels were not affected.

To confirm that these changes were caused by loss of Msl2 we investigated the above modifications in the Msl2-rescue cell line. We found that re-expression of Msl2 restored to almost wild-type levels the defects in modifications observed (Figure 2A, B).

As the MOF-MSL complex is involved in transcriptional regulation in both *Drosophila* and human cells [35], we looked at whether these changes were due to differential transcription of the enzymes thought to be responsible for the modifications. This does not appear to be the case, as no significant difference in the levels of transcript was detected for those tested (*Mof, Suv420* and *Dot1l*) according to Q-PCR (Figure 2C).

### Msl2/hMSL2 Plays a Role in NHEJ

Several of the histone modifications that are affected by loss of Msl2 are implicated in the DNA damage response: H4K16ac [35], [36]; H3K79me2 [11] and H4K20me2 [10], [37]. hMSL2 was shown to co-purify with the NHEJ repair protein DNA-PKcs as part of a human hMOF complex [29]. Moreover, the high expression level of *hMSL2* mRNA in Thymus and T-cells (Supplemental Figure 2) suggests a possible involvement in V(D)J recombination, a process that shares NHEJ machinery [38]. For these reasons we questioned whether Msl2 participates in NHEJ.

**Figure 3:**
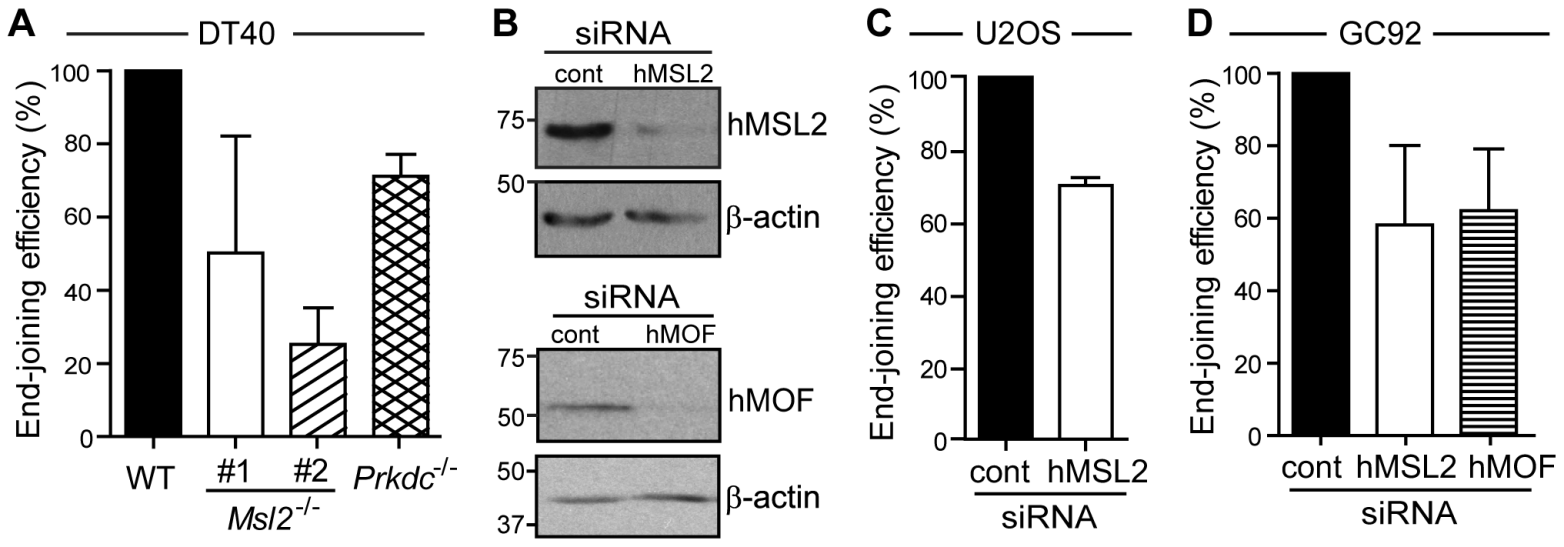
Cells lacking Msl2/hMSL2 have defects in NHEJ repair. (**A**) End-joining efficiency in wild-type, *Msl2^−/−^* and *Prkdc^−/−^* DT40 cell lines as determined by GFP expression measured by flow cytometry analysis, following 16 hour transfection of XmnI-digested GFP plasmid. Transfection efficiency was normalized using uncut GFP plasmid. Repair efficiency was compared to wild-type cells. Compared to wild-type (n = 5): *p* value of *Msl2^−/−^* #1 = 0.0237 (n = 5); *p* value of *Msl2^−/−^* #2 = 0.0586 (n = 3); and *p* value of *Prkdc^−/−^ = *0.0912 (n = 5). (**B**) Representative immunoblot showing hMSL2 (upper two panels) and hMOF (lower two panels) depletion achieved using hMSL2 and hMOF siRNA respectively. (**C**) End-joining repair assay as in (A) in U2OS cells treated with control (cont)- or hMSL2-siRNAs. *p* value = 0.0014 (n = 4). (**D**) NHEJ repair efficiency in GC92 cells treated with control-, hMSL2- or hMOF-siRNAs as determined by repair of I-SceI digested intrachromosomal reporter. Quantified as percentage of CD4 positive cells by flow-cytometry analysis. Efficiency is compared to control cells. p value of hMSL2-siRNA treated cells = 0.0318 (n = 4) and p value of hMOF-siRNA treated cells = 0.1947 (n = 2). Error bars represent standard deviation. Two-tailed Student’s *t*-test was used to generate *p* values.

We first used in vivo end-joining reporter assays, whereby GFP cDNA encoded in a plasmid is blunt-digested with the restriction enzyme XmnI, and transiently transfected into cells. The cell’s ability to re-ligate the broken DNA is measured by the level of GFP protein expressed, as judged by flow cytometry analysis. DT40 cells lacking Msl2 had an impaired ability (∼25–50%) to re-ligate the digested DNA compared to wild-type cells (Figure 3A). A knockout cell line lacking *Prkdc*, the chicken orthologue of *DNA-PKcs* [39], an essential component of the NHEJ repair pathway [40], also showed a decreased ability (71%) to re-ligate the DNA in this assay. As this protein is a crucial factor for NHEJ, we expected a larger defect in this assay for this cell line. We presume that this anomaly is due to limitations in the transfection efficiency in the DT40 system. We therefore wanted to test whether this defect was present in other systems. We first used siRNAs to deplete hMSL2 in U2OS cells. Depletion of the majority of hMSL2 was verified by western blotting analysis using a novel monoclonal antibody raised against a fragment of hMSL2 (Figure 3B, Supplemental Figures 1,3). We then used the assay described above to test the ligation efficiency of these cells. Again we found that cells depleted of hMSL2 had an impaired ability (72%) to repair the digested DNA compared to control siRNA treated cells (Figure 3C).

We also utilized an established intrachromosomal NHEJ substrate-based system [41]; whereby two specific breaks are induced in an integrated reporter cassette by expression of the I-SceI restriction enzyme. Joining of the broken DNA through an NHEJ mechanism can be measured by expression of cell-surface markers using flow cytometry. Depletion of hMSL2 from cells in this assay resulted in a reduction (58%) in the frequency of end-joining compared to control siRNA treated cells (Figure 3D). Interestingly, a similar reduction (62%) was observed in cells that were depleted of hMOF (Figure 3D).

Taken together, these results implicate Msl2/hMSL2 in the NHEJ-mediated repair of DNA damage.

### Msl2/hMSL2 is Stabilized in Response to DNA Damage

**Figure 4:**
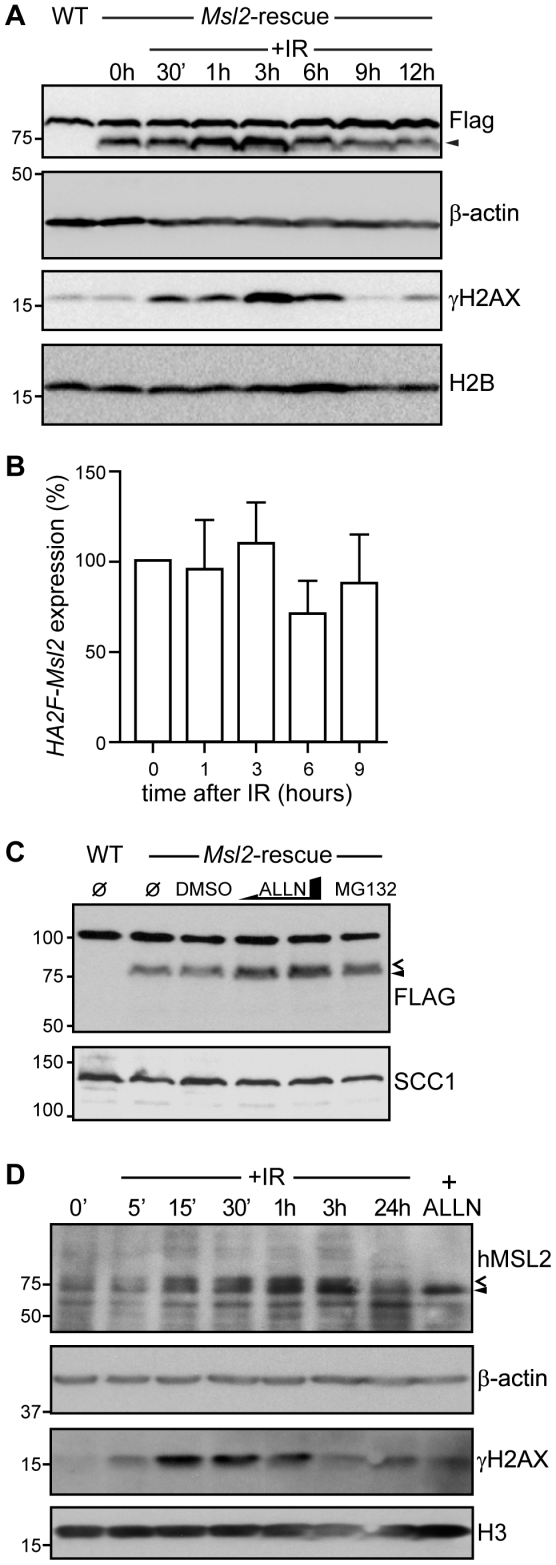
Msl2 and hMSL2 are stabilized following DNA damage. (**A**) Immunoblot analysis of DT40 wild-type and *Msl2*-rescue cells with the antibodies indicated, before and after 5 Gy IR at the times indicated. Arrowhead indicates HA2F-Msl2, the upper band is a non-specific anti-Flag artifact seen in DT40 whole cell extract. (**B**) Q-PCR analysis of the *Msl2*-rescue cell line showing *HA2F-Msl2* expression after 5 Gy IR treatment at the times indicated. Error bars represent standard deviation (n = 3). (**C**) Immunoblot analysis of *Msl2*-rescue cells following treatment with DMSO (vehicle), 50 or 100 µM ALLN or 3 µM MG132 for 8 hours. HA2F-Msl2 is indicated with an arrowhead. Modified HA2F-Msl2 is indicated with an open arrowhead. (**D**) as for (A) except U2OS cells were analysed after 10 Gy IR at the times indicated, or after treatment with 100 µM ALLN for 6 hours. hMSL2 is indicated with an arrowhead. Modified hMSL2 is indicated with an open arrowhead.

An obvious next question to address is how Msl2 behaves in response to DNA damage. In DT40 cells we could not monitor endogenous Msl2, as neither the monoclonal anti-hMSL2 antibody, nor three commercially available antibodies recognize the chicken protein, therefore the Msl2-rescue cell line was used instead. We exposed these cells to 5 Gy of ionizing γ-irradiation (IR), then monitored the cells over a timecourse of 12 hours (Figure 4A). We used the phosphorylation of H2AX on serine 139 (γH2AX) to indicate the DNA damage response over this timecourse; levels begin to increase after 30 minutes, peak after three hours, and return to pre-damage levels by nine hours. Anti-Flag antibody was used to detect the amount of HA2F-Msl2 protein. Interestingly, HA2F-Msl2 protein appeared to accumulate under these conditions; levels begin to increase after one hour and peak at three hours, before returning to pre-damage levels after nine hours. This increase is due to some post-transcriptional effect as there was no significant change in *Msl2* mRNA levels over the timecourse (Figure 4B).

To investigate this stabilization further, we treated the DT40 *Msl2*-rescue cells with the proteasome inhibitors ALLN (N-Acetyl-L-leucyl-L-leucyl-L-norleucinal), and MG132. Both treatments caused an increase in abundance in the amount of HA2F-Msl2 protein as detected by anti-Flag antibody (Figure 4C). Intriguingly, alongside the accumulation of HA2F-Msl2, we noticed the appearance of a band slightly larger than the endogenous protein, suggestive of some post-translational modification.

We next examined endogenous hMSL2 in human U2OS cells. Cells were treated with 10 Gy IR and followed with a timecourse of 24 hours (Figure 4C). Again, we found hMSL2 protein accumulating; beginning 15 minutes after IR, and peaking after three hours. This accumulation followed slightly behind γH2AX detection in this case, which began after five minutes and peaks after 30 minutes. Alongside the accumulation of hMSL2, we again noticed the appearance of a band slightly larger than the endogenous protein. Also in keeping with the DT40 result (Figure 4C) is the accumulation of hMSL2 protein when cells are treated with ALLN (compare first and last lanes, Figure 4C).

These results suggest that soon after, or coincident with the formation of γH2AX foci, Msl2/hMSL2 accumulates in the cell. This accumulation is possibly due to the inhibition of proteolysis of Msl2/hMSL2 by the proteasome, limiting its turnover.

### hMSL2 Mediates Modification of 53BP1 at Lysine 1690

**Figure 5:**
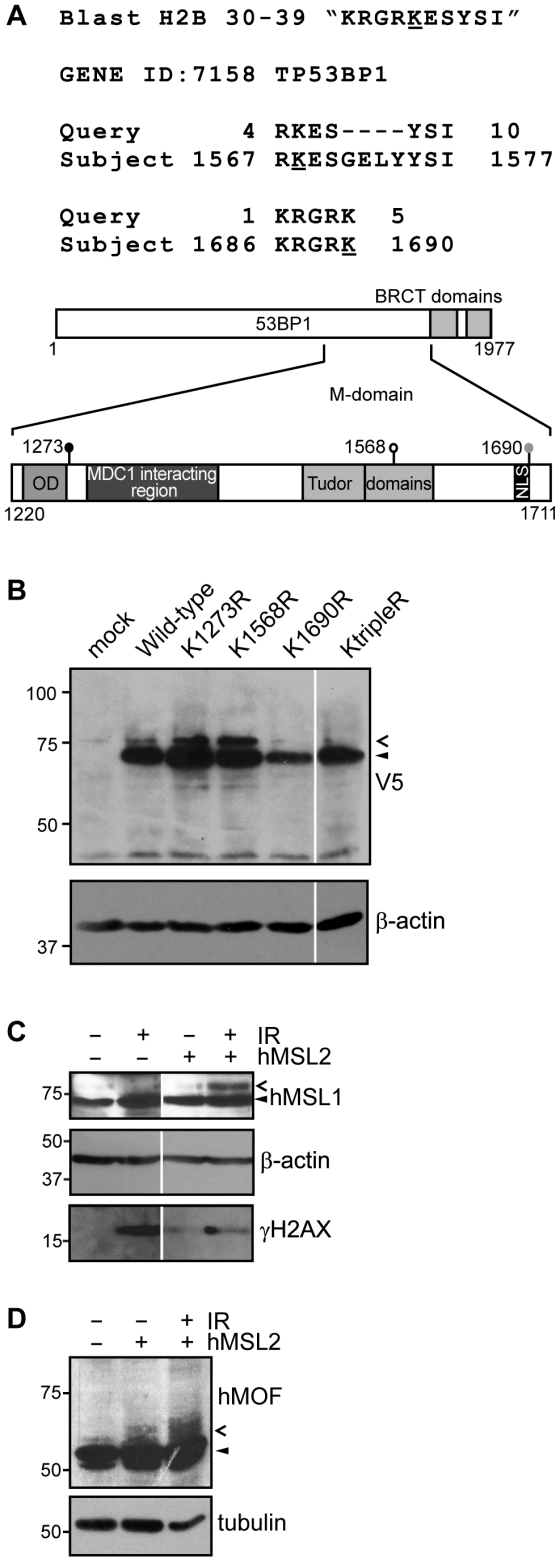
hMSL2 mediates modification of 53BP1, hMSL1 and hMOF. (**A**) Alignment of the two peptide sequences in 53BP1 (subject) that are reported as similar to the sequence containing H2BK34 (query), and schematic showing the M-domain of 53BP1. Lysine 1568 (white circle) lies within the second Tudor domain and lysine 1690 (grey circle) lies within the nuclear localization sequence (NLS). OD represents the oligomerization domain. Also shown is lysine 1273 (black circle) reported to be ubiquitylated by RAD18. (**B**) Immunoblot analysis of U2OS cells that were transfected with both His-ubiquitin and HA2F-hMSL2, together with either V5-53BP1-M-domain wild-type or point mutant constructs. Mock cells were not transfected with any of the plasmids. (**C and D**) Immunoblot analysis of U2OS cells, with transfection of His-ubiquitin and with/without transfection of HA2F-hMSL2. Cells were treated with 10 Gy of IR as indicated and harvested 15 minutes after IR. V5-Mdomain, endogenous hMSL1 and endogenous hMOF are indicated with an arrowhead. Modified proteins are indicated with an open arrowhead.

hMSL2, in cooperation with hMSL1, ubiquitylates histone H2B on lysine 34 (H2BK34ub) [24]. This modification has links with transcriptional regulation, but not with the DNA damage response. In an attempt to identify other DDR-relevant substrates of hMSL2 we blasted the sequence surrounding H2BK34. A number of hits were returned; the first seven relevant (containing the lysine corresponding to K34) protein hits are listed in Table 1. Surprisingly, two of these were located in 53BP1 (Figure 5A). This information prompted us to investigate whether 53BP1 is a substrate of hMSL2.

**Table 1:** List of BlastP hits using H2B peptide as query.

KRGRKESYSI Blast Hit	Gene name	Max identity	E-value
Histone H2B type 3-B	*H2B*	100%	4e-05
Histone H2B type 1-B	*H2B*	90%	5e-04
Histone H2B type 1-M	*H2B*	80%	0.003
Sex comb on midleg-like protein 4	*SCML4*	70%	0.72
Dystonin	*Bpag1*	60%	0.73
Tumor protein p53 binding protein 1	*53BP1*	70%, 50%	1.9, 2.4
Ubiquitously transcribed tetratricopeptide repeat protein Y-linked	*UTY*	50%	2.6

The peptide KRGRKESYSI including K34 (underlined) in histone H2B was used as query in a search for homologous sequences in the human genome using the BLAST online tool. Listed are the first seven hits that include a lysine corresponding to K34. Also shown are their maximal identities and *E*-values.

We used a construct consisting of the minimal domain of 53BP1 required for foci formation (M-domain)(Figure 5A) [42] to generate V5-tagged M-domain constructs containing lysine to arginine point mutations of the residues that correspond to the predicted residues from the blast (K1568 and K1690), as well as point mutation of lysine 1273, known to be ubiquitylated by RAD18 [43]. A triple mutant of these residues was also generated (KtripleR). These constructs were then co-transfected with hMSL2 into U2OS cells for 24 hours and then analysed by immunoblotting analysis. WT, K1273R and K1568R constructs were modified in the presence of exogenous hMSL2 (Figure 5B). However, neither the K1690R construct nor the triple mutant showed the extra band (Figure 5B). We hypothesize that 53BP1 is mono-ubiquitylated at lysine 1690 by hMSL2.

We also wanted to test whether depletion of hMSL2 caused defects in 53BP1 recruitment to damage foci following IR treatment. However, under the conditions used, any differences observed between control and hMSL2-depleted cells were not significant.

### DNA Damage-enhanced Modification of hMSL1 and hMOF via hMSL2

In addition to ubiquitylation of histone H2BK34 [24], it was recently reported that Drosophila MSL2 can ubiquitylate other components of the MSL complex including MOF, MSL1, and MSL3 [18]. We therefore questioned whether hMSL2 could ubiquitylate other members of the human MSL complex, and whether this could contribute to the DNA damage response. U2OS cells were transfected with HA2F-hMSL2, followed by treatment with IR. In the presence of HA2F-hMSL2 we detected an extra band (indicated by open arrowhead) above endogenous hMSL1 (Figure 5C) and hMOF (Figure 5D). This additional band is even more pronounced 15 minutes after treatment with 10 Gy IR. The shift in size is suggestive of mono-ubiquitylation. We therefore hypothesize that both hMSL1 and hMOF are ubiquitylated by hMSL2 in response to DNA damage.

## Discussion

Despite its essential role in *Drosophila* dosage compensation, human MSL2 is relatively poorly characterized. Dosage compensation in mammals is mediated by a different mechanism and by different players [44], so it is likely that hMSL2 has some other function(s). Loss of function approaches using siRNA/shRNA mediated depletion of hMSL2 are difficult; levels of the protein vary between cell lines (Supplemental Figure S3), and depletion requires several rounds of siRNA treatment that does not always achieve acceptable reduced levels of protein [24](and data not shown). To completely ablate Msl2 and better understand its function, we generated and characterised novel DT40 cell lines in which the *Msl2* gene has been disrupted.

These *Msl2* knockout cells appear normal, with only a slight growth defect (Figure 1D). This is surprising in light of our finding that several important histone modifications are disrupted in these cells. However, none of the modifications tested were completely lost, with levels reduced to between ∼25–50% that in wild-type cells (Figure 2B). These residual levels may be sufficient for the cells to grow almost normally. This is also consistent with our proposal of Msl2’s role in the DNA damage response; without damage we would not expect to see a major growth phenotype.

The stabilization of Msl2/hMSL2 in response to damage strongly supports the proposed role of this protein in the DDR (Figure 4). As ALLN/MG132 treatment also leads to hMSL2/Msl2 accumulation, this stabilization appears to be dependent on the avoidance of some proteolytic action against hMSL2/Msl2 that may be keeping it at a basal level.

Using two standard assays to measure NHEJ efficiency [5], we have found that both Chicken Msl2 and human MSL2 contribute to end-joining repair (Figure 3). The requirement of Msl2/hMSL2 for a fully functional NHEJ repair pathway is complex but probably partly converges at the recruitment of 53BP1, a key protein in the NHEJ pathway [5], [6], [7]. 53BP1 is recruited to DNA damage sites by different means: acetylation of H4K16 [28]; di-methylation of H3K79 [8], [11]; and di-methylation of H4K20 [9], [11], [37] have all been shown to (directly or indirectly) recruit 53BP1. We have observed reduced levels of all these modifications (Figure 2) in the *Msl2* knockout cells. The reduction in H4K16ac may be due to lack of MSL complex formation/stability in the absence of Msl2/hMSL2 as previously described in *Drosophila* [16], [45], or, as hMSL2 possibly ubiquitylates hMOF (Figure 5D), as was recently shown in *Drosophila* [46], it is conceivable that this hypothetical ubiquitylation promotes hMOF’s activity towards H4K16. Indeed, as hMOF and H4K16ac are known to have a role in the DNA damage response [28], [29], [30], [31], it is possible that the defects in NHEJ observed here are largely due to aberrant hMOF activity in the absence of hMSL2 regulation.

The reduction in H3K79me2 is probably due to presumed loss of hMSL2 mediated H2BK34 ubiquitylation and the subsequent loss in stimulation of the DOT1L methyltransferase as previously reported [24]. Alongside the reduction in H3K79me2, this last study found reduced H3K4me3 in hMSL2-depleted cells [24]. In yeast, H3K4me3 was demonstrated to be involved in NHEJ [47]. We did not check H3K4me3 levels in our system.

In addition to histone modifications, 53BP1 also gets recruited to damage sites by interaction with the mediator protein MDC1 [48], [49]. Others have previously shown that depletion of hMOF or hMSL1 causes a loss of recruitment of MDC1 to damage foci [28]. It is interesting that both hMSL1 and hMOF appear to get modified in the presence of hMSL2 in response to damage (Figure 5C, D). One could speculate that this modification, possibly ubiquitylation, may regulate these proteins’ interaction with/activity towards MDC1 and somehow promote its recruitment or stabilization at damage sites.

Based on these results in the *Msl2* knockout cells, we expected that 53BP1 recruitment or accumulation would be defective in these cells, so we carried out immunofluorescence microscopy in U2OS cells that had been treated with IR. However, with the conditions and at the timepoints we analysed, we did not observe a significant difference between control- and hMSL2-siRNA treated cells. This may be due to incomplete depletion of hMSL2 in this experiment, whereby the modifications that may recruit 53BP1 are not sufficiently affected.

The modification of 53BP1 on lysine 1690, possibly ubiquitylation, mediated by hMSL2 is interesting (Figure 5B). This lysine residue is part of the nuclear localization sequence. It is noteworthy that Nucleoporin 153 (NUP153), a component of the nuclear pore [50] was recently shown to promote the nuclear import of 53BP1 important for the DDR [51]. Furthermore, NUP153 and hMSL2 are components of the hMSL/hMOF complex [20]. The hypothetical ubiquitylation of this residue could affect 53BP1 interaction with NUP153, and by extension the hMSL/hMOF complex. Alternatively, following initial recruitment to the aforementioned modifications, 53BP1 K1690 ubiquitylation could enhance its oligomerization, or could enhance its interaction with p53 or other proteins/modifications in such a way to promote the accumulation/function of this mediator protein at the site of damage.

We have shown that Msl2/hMSL2 plays a role in NHEJ, but it is possible that Msl2/hMSL2 plays a broader role in regulating the response to DNA damage. Higher levels of damage or unrepaired damage could lead to higher levels of hMSL2, resulting in ubiquitylation of p53, causing its nuclear export and the activation of the mitochondrial-dependent apoptotic pathway previously described [22], [23]. Whereas, in response to low levels of damage we hypothesize that stabilized Msl2/hMSL2 could facilitate the histone/protein modifications described above, promoting the recruitment or accumulation of 53BP1 leading to NHEJ-mediated repair.

## Materials and Methods

### Cell Culture

Previously published DT40 cell lines [39], were provided by Ciaran Morrison (CCB, NUI Galway), and were cultured in RPMI media (Gibco) supplemented with 10% fetal calf serum (Lonza), 1% chicken serum (Sigma-Aldrich), and 1% penicillin/streptomycin (Sigma-Aldrich) at 39.5°C with 5% CO_2_. U2OS cells were commercially obtained from ATCC (American type culture collection), and ST4.5 cells [52] were provided by Rhodri Ceredig (REMEDI, NUI Galway), and both were cultured in DMEM (Dulbecco’s Modified Eagle‘s Medium, Sigma) supplemented with 10% FBS (Fetal Bovine Serum, Sigma and Biosera) at 37°C with 5% CO_2_. Cells were irradiated with gamma rays at the rate of 1294 Gy/hour using a Mainance Millenium ^137^Cs irradiator (Mainance Engineering Ltd). DT40 cells were treated with 50 or 100 µM of ALLN (N-Acetyl-L-leucyl-L-leucyl-L-norleucinal; Calbiochem) or 3 µM MG132 for 8 hours, while U2OS cells were treated with 100 µM of ALLN for 6 hours.

### Generation of *Msl2^−/−^* DT40 Cells

To disrupt the *Msl2* gene, we generated Msl2-puromycin and Msl2-blasticidin disruption constructs by combining two genomic PCR products with the puromycin- or blasticidin-selection-marker cassette. The 2.4 kb targeting arm was amplified by PCR with primers 5′- GATCTGGTACTTTGAGAGCCTGTG-3′ and 5′- CAGATGTGAGTGAACTGCAAGAGAT-3′. The 4.1 kb targeting arm was amplified with primers 5′- actagTTTGAGATGAATTGCTGATGTAAATG-3′ and 5′- acgcgtCAAATGCTGAAGTAGAACTGCTGCA-3′. Amplified PCR products were cloned into pGEM-T easy vector (Promega) and sequenced. To generate *Msl2^−/−^* cells, Msl2-puro and Msl2-bsr disruption constructs lineralized with ApaLI were transfected sequentially by electroporation using the Gene Pulsar electroporation apparatus (Bio-Rad, Wicklow, Ireland). The genomic DNA of the transfectants was digested with BamHI, and gene-targeting events were confirmed by Southern blot using a probe external to the targeting construct. The probe was labeled with digoxigenin (PCR Dig probe synthesis kit, Roche, Germany) and amplified with primers 5′- GGAATGGTGGTGAAGTTTATTACAG-3′ and 5′- CTAACCCATCCTCAAACCCAAG-3′.

### Cloning of Msl2 cDNA and Generate Stable DT40 Msl2^−/−^ Rescue Cell Line

Chicken Msl2 cDNA was isolated by PCR amplification of the primary cDNAs using the 5′-ctcgagctATGAACCCGGTGAATGCCA-3′ and 5′-gaattcTCAACAGTCATATCTCACGTCTATAGCT-3′ primers. The gene bank accession number of the chicken *Msl2* gene is XM_426675. The PCR fragment was digested by XhoI and EcoRI and inserted into modified pCDNA3.1-HA-2xFlag vector. The result plasmid was used to generate stable DT40 Msl2^−/−^ rescue cell line.

### Proliferation Analysis

For cell proliferation analysis, cultures were seeded in 24-well plates in triplicate at equal cell densities (5×10^4^ cells/ml) and counted every 24h up to 96h.

### Flow Cytometry Analysis

Cells were harvested, fixed with 70% ethanol. For mitotic index determination, cells were treated with rabbit anti-H3ser10ph monoclonal antibody and subsequently with fluorescein isothiocyanate-conjugated anti-rabbit IgG antibody (Jackson Immunoresearch). The cells were resuspended in phosphate-buffered saline containing propidium iodide at 25 ug/ml and RNase-A at 250 ug/ml. The subsequent FACS analysis was performed with a FACS Canto apparatus and FACS Diva software (Becton Dickinson). For the analysis of the NHEJ assay, cells were washed in 1X PBS and DT40 cells were treated with 10 U of DNAseI for 15 minutes at room temperature. 5×10^5^ of U2OS cells or 1×10^6^ of DT40 cells were analysed for GFP expression using a FACS Canto (Becton Dickinson) and analysed using the BD FACS Diva Software (version 6.1.2, Becton Dickinson).

### Quantitative Real-time PCR

Total RNA was obtained from DT40 cell lines using the ISOLATE RNA mini kit (Bioline) and reverse transcribed using High capacity RNA-to-cDNA kit (Applied Biosystems), according to the manufacturers’ guidelines. cDNA was quantified following quantitative real-time PCR with primers against *Msl2*, *Mof*, *Dot1l*, *Suv420*, *Gapdh*, *β-Actin* using fast SYBR green master mix (Applied Biosystems) in a ABI 7500 fast (Applied Biosystems) according to manufacturers guidelines. Sequences of the real-time primers used are available upon request.

### Antibodies and Immunoblotting

Whole-cell extracts were prepared with RIPA-buffer (50 mM Tris-HCl, pH 7.4, 1% NP-40, 0.25% sodium deoxycholate, 150 mM NaCl, 1 mM EDTA, and protease inhibitor cocktail). Nuclear and cytoplasmic fractions were obtained as previously described [53]. Proteins were transferred to nitrocellulose/PVDF membranes for analysis using the following primary antibodies: anti-H4K16ac (07–329, Upstate), anti-H4K20me2 (ab14964, Abcam), anti-H4K20me3 (07–463, Upstate), anti-H3K9me2 (# 4658P, Cell Signaling Technology), anti-H3K79me2 (ab3594, Abcam), anti-H3ser10ph (06–570, Millipore), anti-Flag (F1804, Sigma), anti-beta-actin (ab8227, Abcam), anti-SCC1 [54], anti-alpha-tubulin (T 6074, Sigma) anti-gamma-H2AX (05–636, Millipore), anti-H3 (ab1791, Abcam), anti-V5 (MCA1360, AbD Serotec), anti-hMSL1 (Akhtar lab). Anti-hMOF (7D1) & anti-hMSL2 (4F12) mouse monoclonals: GST-hMOF (full-length) and GST-hMSL2-fragment (residues 86–412) were used as antigen. Secondary antibodies: anti-mouse HRP (NA931, Amersham) and anti-rabbit-HRP (NA934, Amersham). For densitometry quantification, chemiluminescence was detected using a Fujifilm LAS300 (Fujifilm) and quantified using ImageJ software.

### siRNA Transfection

1×10^5^ cells in a 6 well dish were treated with 10nM siRNA, 4 µl of oligofectamine (Invitrogen), in 1.5ml OptiMem (Invitrogen) for 18 hours. For hMSL2 depletion, cells received 3 rounds of treatment over 6–7 days. For hMOF, hMSL1 or hMSL3 depletion, cells were treated once for 2–3 days. The siRNAs used in this paper are as follows: control: siGENOME RISC-Free Control (Dharmacon, sequence unavailable); hMSL2: ON-TARGETplus SMARTpool GUGUAAUGGCAGCGAAACA+ AAACAUCAUAUGCCGGAAA+ CACCAUGCCUCCCGAAAUU+ GUGUCAAAUUGGAGGGUAA (Dharmacon), hMOF:GGAAAGAGAUCUACCGCAA [dT][dT] (Sigma);

### *In vivo* NHEJ Assay

U2OS cells were pre-treated siRNA, then 2×10^5^ cells/well were seeded in a 6-well plate 24 hours before the assay. DT40 cells were grown until confluency (1×10^6^ cells/ml) and 1×10^6^ cells were used for the assay. U2OS cells were transfected with 1 µg of uncut pmaxGFP plasmid (Lonza) or with 1 µg of pmaxGFP plasmid linearized by restriction digest with XmnI enzyme within the GFP sequence using the Lipofectamine 2000 (Invitrogen). DT40 cells were electroporated with the same plasmids using the Amaxa nucleofection system and program B-23 (Amaxa). U2OS cells were harvested 24 hours post-transfection and DT40 cells 16 hours post-electroporation and analysed by flow cytometry. Following knockdown, the *in vivo* NHEJ ligation assay in the GC92 cell line was performed as described previously [55].

### Plasmid Transfection

U2OS cells were seeded in a 6-well plate (2×10^5^ cells/well) 24 hours prior to plasmid transfection. Cells were transfected with 1 µg of indicated plasmids and cells were harvested 48 hours post-transfection directly in 2X Laemmli buffer. If subjected to IR treated, cells were harvested 15 minutes post-irradiation.

## Supporting Information

Figure S1 **Alignment of human hMSL2 and chicken Msl2.** Msl2 was aligned with hMSL2 using bl2seq on NCBI. The RING domain is boxed in green, the CXC domain in red. A construct comprising residues 86 to 412 (sequence between RING and CXC domains) was used in the generation of the monoclonal anti-hMSL2 antibody.
(TIF)

Figure S2 ***hMSL2*** **mRNA expression profile.** Expression of *hMSL2* mRNA in 79 human tissues according to the Affymetrix Human U133A chip as analysed using the online bioinformatic tool www.biogps.org [56], [57].
(TIF)

Figure S3 **Generation and characterization of hMSL2 monoclonal antibody.** (**A**) Coomassie stained gel showing induction and purification of 6His-hMSL2 fusion construct from *Escherishia coli.* 6His-hMSL2 comprises amino acids 86 to 412 of hMSL2 and has a predicted molecular weight (MW) of 45 kDa. (**B**) Immunoblot analysis of whole cell extract from HeLa cells transfected with empty plasmid (mock) or a plasmid encoding HA-2F-hMSL2. Antibodies (hMSL2 hybridoma supernatent number) used are indicated below the blot. Endogenous hMSL2 has a predicted molecular weight of 75 kDa, and HA-2F-hMSL2, 80 kDa. 8A4, 4F12 and 8D2 correspond to different hybridoma supernatents tested. (**C**) Immunoblot analysis of U2OS cells transfected with siRNA against hMSL2 or with plasmids encoding HA-2F-hMSL2 or hMSL2-YFP (hMSL2 C-terminally-tagged with yellow fluorescent protein; MW 100 kDa). ST4.5 is a T-cell progenitor cell line [52].
(TIF)
