# Supplementary figures and images for: Msl2 Is a Novel Component of the Vertebrate DNA Damage Response
Source: PLoS One. 2013 Jul 9;8(7):e68549. doi: 10.1371/journal.pone.0068549 (PMC3706407; doi:10.1371/journal.pone.0068549)

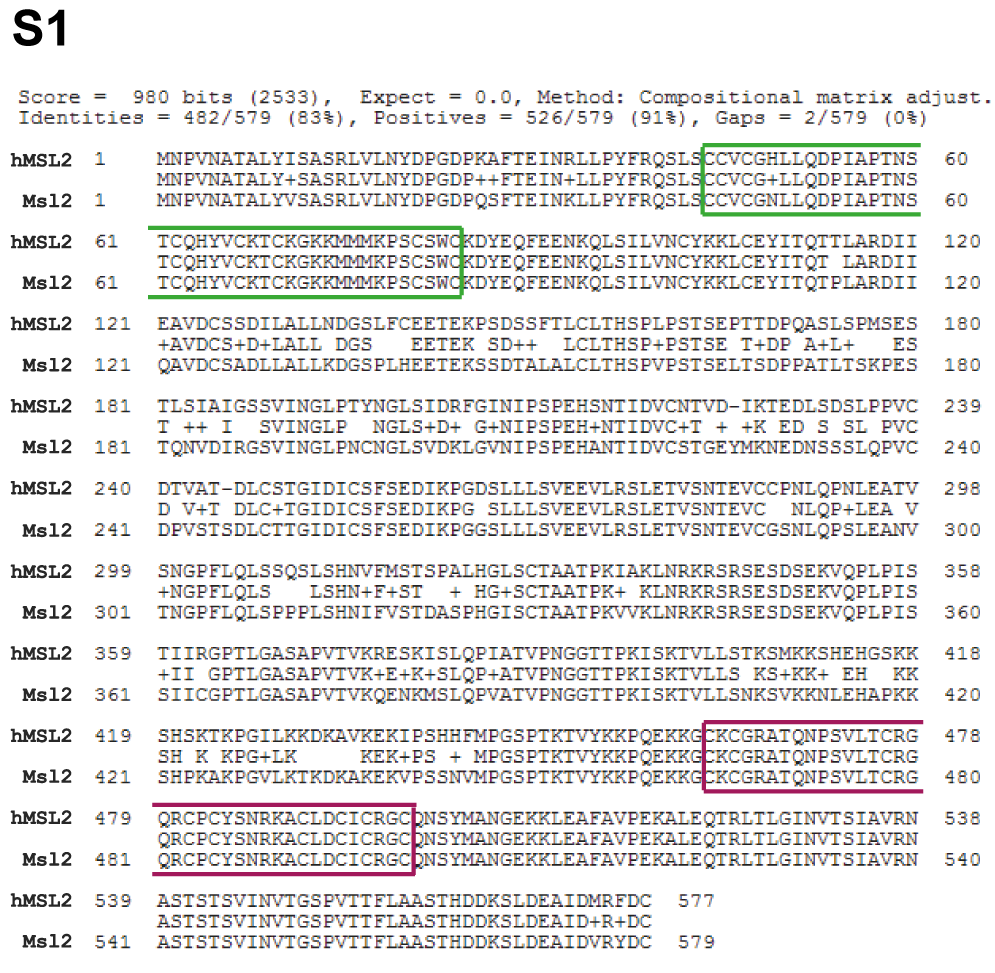

Supplement: Figure S1 — Alignment of human hMSL2 and chicken Msl2. Msl2 was aligned with hMSL2 using bl2seq on NCBI. The RING domain is boxed in green, the CXC domain in red. A construct comprising residues 86 to 412 (sequence between RING and CXC domains) was used in the generation of the monoclonal anti-hMSL2 antibody. (TIF) [file pone.0068549.s001.tif]

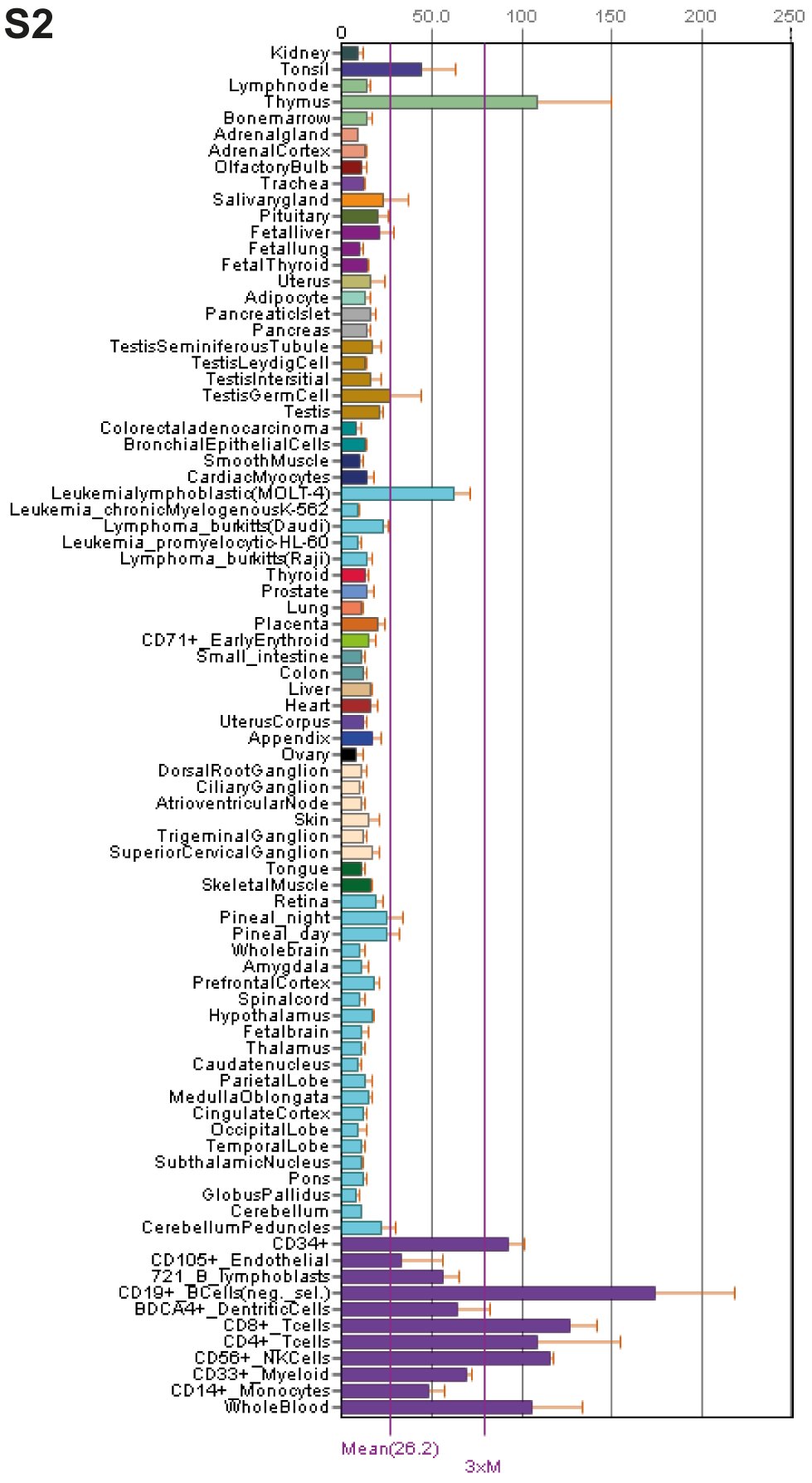

Supplement: Figure S2 — hMSL2 mRNA expression profile. Expression of hMSL2 mRNA in 79 human tissues according to the Affymetrix Human U133A chip as analysed using the online bioinformatic tool www.biogps.org [56], [57]. (TIF) [file pone.0068549.s002.tif]

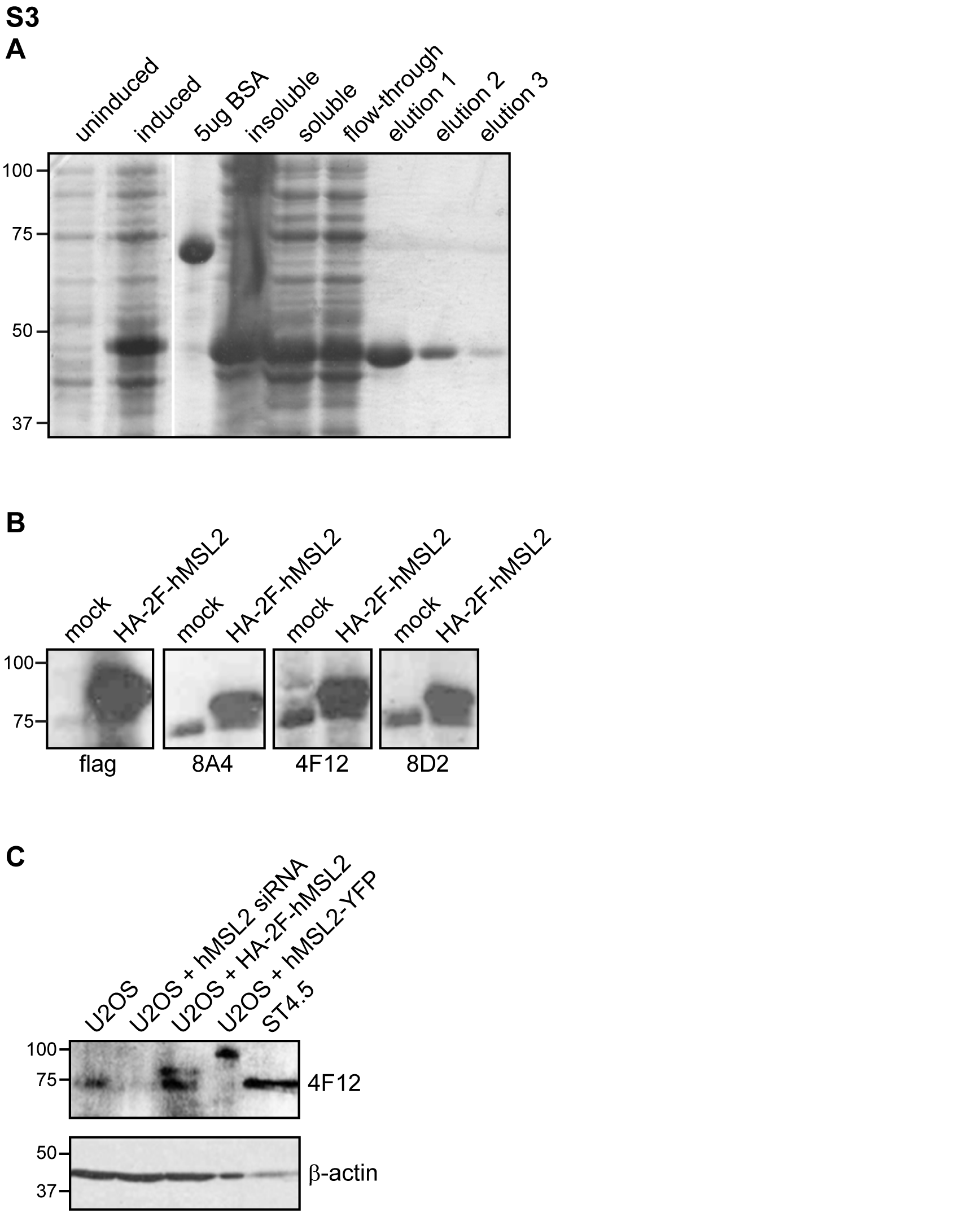

Supplement: Figure S3 — Generation and characterization of hMSL2 monoclonal antibody. (A) Coomassie stained gel showing induction and purification of 6His-hMSL2 fusion construct from Escherishia coli. 6His-hMSL2 comprises amino acids 86 to 412 of hMSL2 and has a predicted molecular weight (MW) of 45 kDa. (B) Immunoblot analysis of whole cell extract from HeLa cells transfected with empty plasmid (mock) or a plasmid encoding HA-2F-hMSL2. Antibodies (hMSL2 hybridoma supernatent number) used are indicated below the blot. Endogenous hMSL2 has a predicted molecular weight of 75 kDa, and HA-2F-hMSL2, 80 kDa. 8A4, 4F12 and 8D2 correspond to different hybridoma supernatents tested. (C) Immunoblot analysis of U2OS cells transfected with siRNA against hMSL2 or with plasmids encoding HA-2F-hMSL2 or hMSL2-YFP (hMSL2 C-terminally-tagged with yellow fluorescent protein; MW 100 kDa). ST4.5 is a T-cell progenitor cell line [52]. (TIF) [file pone.0068549.s003.tif]
